# Rapid Identification of Trace Pharmacodynamic Substances in Traditional Chinese Medicine via SERS and Deep Learning

**DOI:** 10.3390/bios16030139

**Published:** 2026-02-27

**Authors:** Huixuan Yang, Mingyuan Chen, Jiayi Wang, Xufei Tong, Huiru Li, Hao Chen, Zengshan Yu, Chunying Zhao, Mingli Wang, Guochao Shi

**Affiliations:** 1 Hebei International Research Center for Medical-Engineering, Chengde Medical University, Chengde 067000, China; 15613623894@163.com (H.Y.); 18631451103@163.com (X.T.); 13373088018@163.com (H.L.); 17320970047@163.com (H.C.); 19831488343@163.com (Z.Y.); 2 Hebei Key Laboratory of Study and Exploitation of Chinese Medicine, Chengde Medical University, Chengde 067000, China; 15731626635@163.com; 3 School of Nursing, Chengde Medical University, Chengde 067000, China; wjy082805@163.com; 4 State Key Laboratory of Metastable Materials Science and Technology, Key Laboratory for Microstructural Material Physics of Hebei Province, School of Science, Yanshan University, Qinhuangdao 066004, China

**Keywords:** surfaced-enhanced Raman scattering, Ag nanoparticles, deep learning, traditional Chinese medicine, pharmacodynamic substance

## Abstract

In the modernization of traditional Chinese medicine (TCM), trace detection of pharmacodynamic substances faces a critical challenge: insufficient sensitivity, which significantly hinders accurate quality assessment and standardization. Conventional techniques often fail to measure trace components in complex sample matrices. Therefore, the development of a rapid, effective, sensitive, and reliable analytical method, along with a corresponding quality evaluation system, is of great importance. This study used moth wing (MW) scales as a template to fabricate an Ag/MW SERS substrate via magnetron sputtering. The optimal Ag_30_/MW SERS substrate (30 min sputtering) achieved an enhancement factor of 6.47 × 10^6^ and good reproducibility (minimum RSD: 7.03%). Principal component analysis (PCA) was integrated with four deep learning algorithms (MLP, Transformer, ResNet, DNN) to detect three typical TCM pharmacodynamic substances in pure standard solutions: atractylon, cimifugin, and timosaponin A-III. The models enabled rapid identification, with the MLP model reaching 95.00% accuracy. This research provides a novel, highly accurate, and efficient detection method with potential for TCM pharmacodynamic substances, demonstrating feasibility for bioactive compound identification in model systems, and shows promising potential for future application in TCM composition analysis and quality control.

## 1. Introduction

Traditional Chinese medicine (TCM) has long played a significant role in preventing and treating human diseases [1]. However, the complex composition of TCM makes the detection of its pharmacodynamic substances critically important yet challenging. While advanced techniques like ultra-performance liquid chromatography and liquid chromatography-mass spectrometry are commonly employed for analyzing trace pharmacodynamic substances, they are often limited by complex operational procedures, long processing times, and insufficient sensitivity for trace-level detection in complex matrices [2]. Therefore, there is an urgent need for a rapid, effective, sensitive, and reliable analytical method, along with a corresponding quality evaluation system, for TCM analysis. Surface-enhanced Raman scattering (SERS) has emerged as a highly promising analytical technique for this purpose. Capable of single-molecule detection and providing detailed molecular fingerprint information, SERS offers a powerful tool for identifying trace pharmacodynamic substances [3,4]. Its high sensitivity, reliability, and non-destructive nature have led to its widespread application in fields such as environmental monitoring and food safety [4].

In recent years, SERS has garnered increasing interest for analyzing complex botanical and medicinal samples due to its ultra-sensitive detection and molecular fingerprinting capabilities [5]. For instance, Li et al. employed a bifunctional TiO_2_@Ag core–shell superstructure as a SERS-sensing platform to successfully identify specific flavonoids in a complex Chinese herbal medicine extract, demonstrating the technique’s selectivity [6]. Similarly, the natural grating-like microstructure of moth wings has been effectively utilized as a template for fabricating tunable and cost-effective SERS substrates [7]. Despite these advances, detecting trace pharmacodynamic substances within complex TCM matrices remains a significant hurdle. While recent studies have integrated SERS with machine learning for applications like geographic origin identification of *Morus alba* Linn. [8] or the analysis of herbal decoctions [9], many high-performance SERS substrates still rely on complex or costly fabrication techniques, limiting their practical scalability for routine TCM analysis [10]. Furthermore, the practical application of SERS is often constrained by challenges such as substrate fabrication complexity, poor signal stability, and the difficulty of interpreting complex, high-dimensional spectral data [11,12,13]. Traditional analytical methods have inherent limitations in identifying subtle feature variations within such complex data. In contrast, deep learning algorithms, with their robust feature-learning capabilities, offer significant advantages by enabling automatic extraction of discriminative features from complex Raman spectra, thereby overcoming the limitations of manual interpretation [14,15].

To address these challenges, this study proposes an integrated analytical strategy that combines a cost-effective, bio-inspired SERS substrate with a deep learning framework. The substrate design is informed by prior work utilizing natural microstructures as efficient SERS templates [7,16,17,18]. Concurrently, deep learning models are employed for automated feature extraction and classification, aiming to enhance the accuracy and efficiency of spectral interpretation for precise identification of target substances, particularly in cases of peak overlap or subtle spectral variations [19].

**Figure 1:**
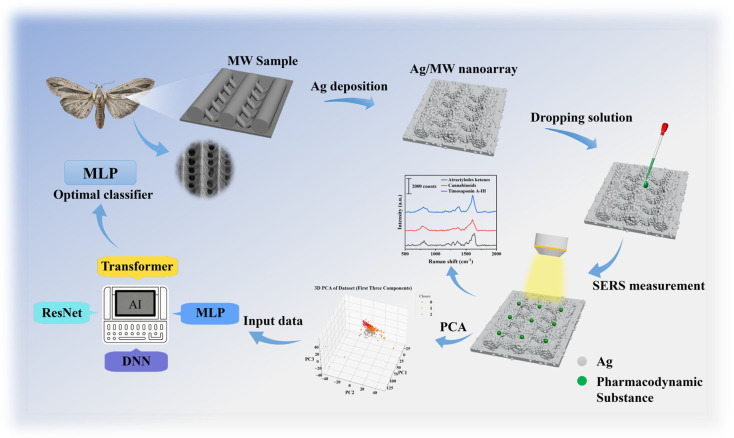
Schematic of the fabrication process for Ag/MW SERS substrates and their application in the Raman spectroscopic detection of pharmacodynamic substances.

As illustrated in Figure 1, this study tackles the issue of insufficient sensitivity in trace detection of TCM active constituents by developing a novel SERS substrate. Using the natural grating-like micro-nano structure of a moth wing (MW) as a template, an Ag/MW nanoarray was fabricated via magnetron sputtering. To achieve rapid, accurate, and quantitative analysis, deep learning algorithms—including Multi-Layer Perceptron (MLP), DNN, Transformer, and ResNet architectures—were employed to process and classify the complex spectral data, ultimately identifying the optimal classifier for distinguishing pharmacodynamic substances. Based on this integrated platform, we successfully achieved trace detection of three key TCM pharmacodynamic substances: atractylon, cimifugin, and timosaponin A-III. This work not only provides a new pathway for detecting TCM ingredients but also offers significant theoretical and experimental support for advancing related analytical methods and strategies.

## 2. Experimental Section

### 2.1. Materials and Instrumentation

The silver target for magnetron sputtering (60.0 mm in diameter, 2.0 mm in thickness, 99.99% purity) was purchased from Nanchang Hanchen New Material Technology Co., Ltd., Nanchang, China. Atractylon, cimifugin, and timosaponin A-III were purchased from Chengdu Nakeli Biotechnology Co., Ltd., Chengdu, China. Moth wings were purchased from Beijing Jiayingshengda Biotechnology Co., Ltd., Beijing, China. Rhodamine 6G (R6G) solution was purchased from Beijing Jiakai Science and Technology Co., Ltd., Beijing, China. Absolute ethanol was purchased from Tianjin Open Chemical Reagent Co., Ltd., Tianjin, China. Deionized water was used to prepare all solutions in the experiments.

The high-vacuum magnetron sputtering system (Shenyang RF Scientific Instruments Co., Ltd., Shenyang, China) was used for depositing silver films. The ion beam composite film deposition system (Model FJL560, Shenyang Scientific Instruments Co., Ltd., Shenyang, China) was employed for substrate pretreatment and composite film fabrication. The surface morphology and microstructure of the SERS substrates were characterized using field emission scanning electron microscopy (FE-SEM, Model FEI-NOVA NANOSEM 230, FEI Company, Hillsboro, OR, USA). All Raman spectroscopic measurements were performed using a Raman spectrometer (Model DXR2xi, Thermo Fisher Scientific Inc., Waltham, MA, USA). A 532 nm laser was used as the excitation source, with an exposure time of 10 s and an incident power of 10 mW for spectral acquisition.

Data processing and analysis were conducted using Origin (Version 10.10.178). Particle size distribution analysis was performed with Nano Measurer (Version 1.2). Deep learning models were implemented using PyCharm Community Edition (Version 242.23339.19). Schematic illustrations were created with Autodesk 3ds Max (Version 27.1.0.11275).

### 2.2. Preparation of Ag/MW Substrates

Before the experiment, the MWs were soaked and cleaned in anhydrous ethanol to remove surface contaminants, then dried and stored at room temperature. In the magnetron sputtering experiments, a high-vacuum magnetron sputtering system and an ion beam composite film deposition system were used to prepare the substrates. First, the silver target was fixed on the sputtering target, and the MW was placed on the sample stage inside the sputtering chamber. Then, the mechanical pump and the compound vacuum gauge were activated. The system was allowed to evacuate until the pressure reached 10 Pa. After starting the molecular pump and the rotary pump, the Power A switch was turned on for further evacuation until the pressure in the sputtering chamber reached 3.5 × 10^−3^ Pa. Next, the function key of the compound vacuum gauge was adjusted until the manual indicator lit up. When the flow indicator light turned on, the MFC2 valve was set to 180 mL/min. The sputtering power was set to 100 W. Once magnetron sputtering began, the Ag atoms from the target were continuously bombarded onto the substrate surface. The sputtering time was adjusted to 10, 20, 30, and 40 min, respectively. By controlling the sputtering time, we successfully fabricated Ag/MW substrates with the same material but different deposition durations. These SERS substrates are denoted as Ag_x_/MW, where x represents the magnetron sputtering time in mins.

### 2.3. Preparation of Probe Molecule R6G and Pharmacodynamic Substance Solutions

A ten-fold serial dilution method was employed to prepare R6G solutions at different concentrations using deionized water. A 10 μL aliquot of each solution was deposited onto the prepared SERS substrate, allowed to dry at room temperature, and then subjected to SERS analysis. Atractylon, cimifugin, and timosaponin A-III were dissolved in deionized water. A 10 μL volume of each pharmacodynamic substance solution at the same concentration was separately dropped onto the prepared SERS substrates. After complete drying at room temperature, Raman spectral analysis was performed.

## 3. Results and Discussion

### 3.1. Morphology Characterization

**Figure 2:**
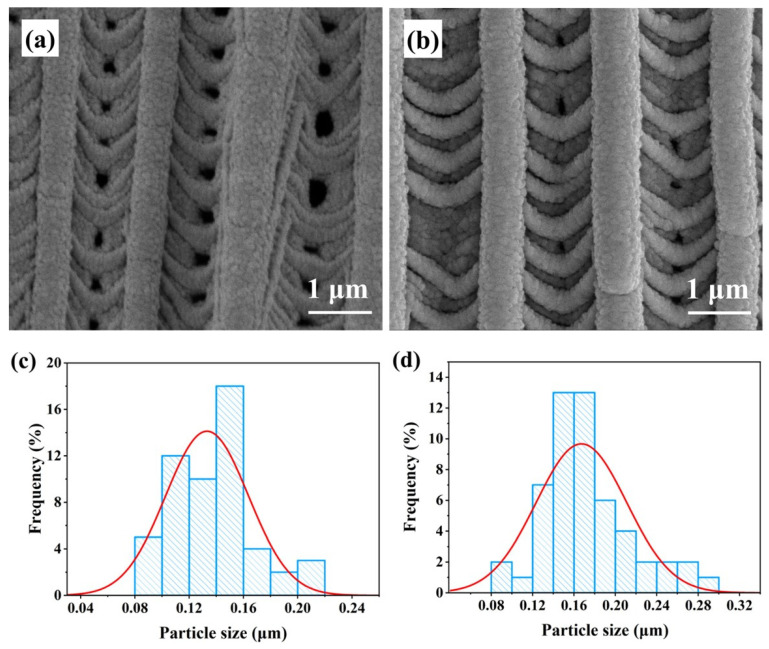
(**a**,**b**) FE-SEM characterization of samples with different sputtering durations. (**c**) Particle size distribution of Ag nanoparticles in Ag_20_/MW. (**d**) Particle size distribution of Ag nanoparticles in Ag_30_/MW.

The surface of the gray moth wing exhibits numerous regular grating-like structures, each consisting of a groove and a ridge. The pristine moth wing substrate comprises multiple parallel grating structures with uniform width and spacing. To construct a plasmonic nanoplatform, we modified the moth wing substrate surface with noble metal Ag nanoparticles using magnetron sputtering technology [20]. Figure 2a,b show field-emission scanning electron microscopy (FE-SEM) images of the Ag_20_/MW and Ag_30_/MW substrates, respectively. As the sputtering time increased, the surface roughness gradually increased while the overall regular arrangement was maintained. To analyze the Ag nanoparticle size distribution, 50 particles were randomly selected from the FE-SEM images of each substrate. As shown in Figure 2c, the diameters of Ag nanoparticles on the Ag_20_/MW SERS substrate range from 0.1 to 0.2 μm, accounting for 85.19% of the measured particles. Figure 2d indicates that the diameters of Ag nanoparticles on the Ag_30_/MW SERS substrate also fall within 0.1–0.2 μm, representing 86% of the counted particles. These uniformly distributed Ag nanoparticles ensure a high density of SERS “hot spots” on the substrate.

### 3.2. Selection of the Optimal Substrate

**Figure 3:**
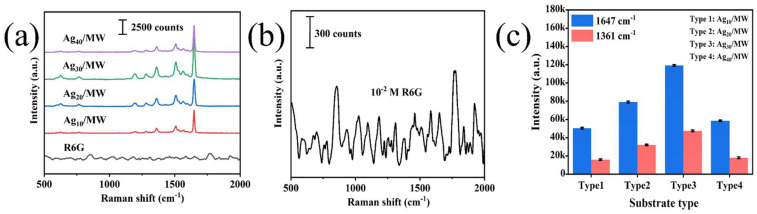
(**a**) SERS spectra of 1 × 10^−6^ M R6G solution on four different substrates; (**b**) Raman spectra for R6G on MW, without Ag; (**c**) Raman peak intensities at characteristic bands at 1361 cm^−1^ and 1648 cm^−1^ across the four substrates.

The performance of the SERS substrate critically influences the success and effectiveness of SERS experiments. Therefore, screening substrates with excellent Raman signal enhancement capability is a key prerequisite for ensuring experimental reliability and detection accuracy. Among the many factors influencing Raman signal intensity, the enhancement factor of the noble metal nanomaterials, the type of nanostructure of the substrate itself, and its surface roughness are particularly important. These conditions directly affect the signal amplification efficiency during detection, thereby determining the overall detection sensitivity. For this reason, we used Rhodamine 6G (R6G) as a standard probe molecule [21] to systematically compare the Raman spectral responses of different substrates. The prominent characteristic peaks of R6G are located at 611 cm^−1^, 774 cm^−1^, 1187 cm^−1^, 1361 cm^−1^, 1510 cm^−1^, 1572 cm^−1^, and 1648 cm^−1^, with the corresponding vibrational modes listed in Table 1 [16]. Figure 3a presents the SERS spectra of a 1 × 10^−6^ M R6G solution acquired on four different Ag_x_/MW substrates (x = 10, 20, 30, 40 min sputtering time). In contrast, Figure 3b shows the Raman spectrum for R6G on the pristine MW substrate without any Ag coating, revealing only very weak signals, which underscores the essential role of the deposited silver nanoparticles for signal enhancement. A significant enhancement in Raman signal intensity was observed for all Ag-coated substrates compared to the bare MW, indicating that the silver nanoparticles effectively amplify the electromagnetic field strength on the substrate surface via the localized surface plasmon resonance (LSPR) effect. For a quantitative comparison, the distinct peaks at 1361 cm^−1^ and 1648 cm^−1^ were selected. Figure 3c compares the Raman signal intensities at these two characteristic peaks across the four Ag-coated substrates. The results demonstrate that the Ag_30_/MW SERS substrate (30 min sputtering) exhibited superior signal intensity at both peaks compared to the substrates with shorter or longer deposition times. This optimal performance is attributed to the dense and uniform deposition of Ag nanoparticles achieved at this sputtering duration, which maximizes the density of SERS “hot spots” and enhances LSPR. Consequently, the Ag_30_/MW substrate was selected as the optimal platform for all subsequent experiments.

### 3.3. Calculation of the Enhancement Factor for the SERS Substrate

In the trace detection of TCM pharmacodynamic substances, the enhancement capability of the SERS substrate is central to detection performance [9]. In this study, using the MW with its naturally occurring grating-like micro-nano structure as a biological template, a series of Ag/MW SERS substrates were fabricated via magnetron sputtering technology. The Ag_30_/MW substrate, prepared with a sputtering time of 30 min, was identified as the optimal choice following a performance-based screening process. Its enhancement factor (EF) was calculated as follows:

The Ag_30_/MW substrate, adsorbed with a 1 × 10^−8^ mol/L R6G solution, served as the SERS substrate, while a flat silicon wafer adsorbed with a 1 × 10^−2^ mol/L R6G solution was used as the reference substrate [23]. The EF was calculated according to the following equation [24]:

$$EF=\frac{I_{SERS}/N_{SERS}}{I_{Raman}/N_{Raman}}$$

where *I*_SERS_ and *I*_Raman_ are the Raman signal intensities at the characteristic peak of 1648 cm^−1^ for the substrate adsorbed with 10^−8^ mol/L R6G solution and the substrate adsorbed with 10^−2^ mol/L R6G solution, respectively. *N*_SERS_ and *N*_Raman_ represent the numbers of R6G molecules on the SERS substrate and the silicon wafer substrate, respectively. Their ratio is calculated using the following formula [24]:

$$N=\frac{N_{A}\times M\times V_{\mathrm{solution}}}{S_{\mathrm{sub}}}\times s_{\mathrm{laser}}$$

In the formula, *N*_A_ is Avogadro’s constant, *M* is the molar concentration of the R6G solution, *V*_solution_ is the volume of R6G solution used in the experiment (10 μL), *S*_laser_ is the laser spot area (0.785 μm^2^), and *S*_sub_ is the adsorption area of R6G on the substrate. Experimental measurements showed that the number of R6G molecules on the reference substrate was about 1.2 times that on the Ag_30_/MW SERS substrate. The final calculation results indicate that the enhancement factor of the Ag_30_/MW SERS substrate reached 6.47 × 10^6^. This high enhancement performance originates from the dense SERS “hot spots” created by the uniformly distributed Ag nanoparticles on the substrate surface (particle size 0.1–0.2 μm, accounting for 86%), which significantly amplify the Raman signal through the localized surface plasmon resonance (LSPR) effect [25].

### 3.4. Time Stability and Reproducibility Test

**Figure 4:**
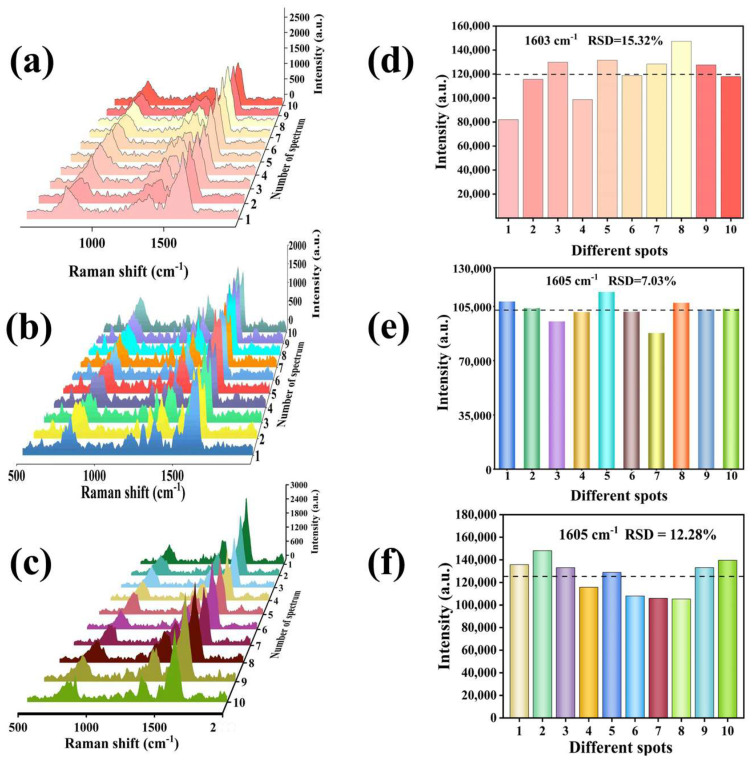
Raman analysis of three bioactive compounds at different substrate positions: (**a**–**c**) Representative Raman spectra of atractylon, cimifugin, and timosaponin A-III at varying substrate locations, each at a concentration of 1 × 10^−6^ M. (**d**–**f**) The RSD results at the corresponding different peak positions.

**Figure 5:**
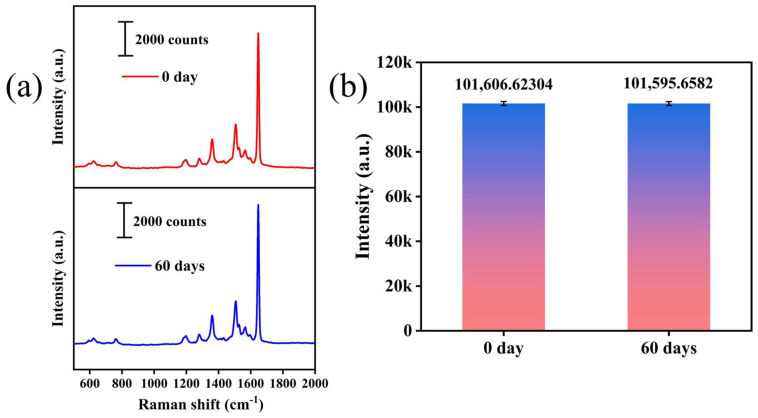
(**a**) Raman signal intensities of 1 × 10^−5^ M R6G solution on Ag_30_/MW SERS substrates at 0 and 60 days; (**b**) bar charts depicting the Raman signal variations in the TCM solutions sputtered on Ag_30_/MW SERS substrates after 0 and 60 days.

Furthermore, the reproducibility of the Ag_30_/MW SERS substrate meets practical application requirements. In the field of TCM pharmacodynamic substance detection, traditional SERS spectral analysis relies on manual feature extraction. When dealing with multi-component systems such as atractylon, cimifugin, and timosaponin A-III, whose characteristic peaks are prone to overlap, this approach is not only inefficient but also susceptible to identification errors due to subjective judgment [26]. Therefore, we used the Ag_30_/MW SERS substrate to detect the Raman spectra of these three pharmacodynamic substances. To ensure data diversity, 10 spectra were collected for each substance at different positions on the substrate. The results shown in Figure 4 exhibit good reproducibility. The relative standard deviation (RSD) of the Raman signal was only 7.03%, indicating good uniformity of the nanostructure on the substrate surface and low signal fluctuation. As shown in Figure 5, we compared the Raman signal intensities of 1 × 10^−5^ M R6G solution on the Ag_30_/MW SERS substrate immediately after preparation (0 days) and after 60 days and found that the signal remained strong even after 60 days, demonstrating good short-term signal retention under controlled laboratory conditions. In summary, with its low detection limit, high enhancement factor, excellent reproducibility, and promising short-term stability, the Ag_30_/MW SERS substrate provides a reliable platform for the ultrasensitive detection of pharmacodynamic substances in TCM.

### 3.5. Identification of Pharmacodynamic Substance in TCM via Deep Learning Combined SERS Technology

The Raman spectra of the three pharmacodynamic substances detected by the Ag_30_/MW SERS substrate served as the original data. To ensure the representativeness and independence of the spectral data, all measurements were performed at different positions on the substrate rather than through repeated sampling at identical spots, 100 spectra were collected for each of the three pharmacodynamic substances to ensure data diversity. The combined dataset for the three TCM substances was then split into a training set and a test set in an 80% to 20% ratio.

Prior to modeling, spectral preprocessing was conducted to enhance signal quality. A Savitzky–Golay filter (second-order polynomial, optimized window size) was applied for smoothing to suppress high-frequency noise while preserving peak shapes. Subsequently, baseline correction was performed using the adaptive iteratively reweighted penalized least squares (AirPLS) method (baseline smoothing parameter: 100, difference order: 1, maximum iterations: 15). The fitted baseline was subtracted to obtain higher-quality SERS spectra for subsequent analysis.

**Figure 6:**
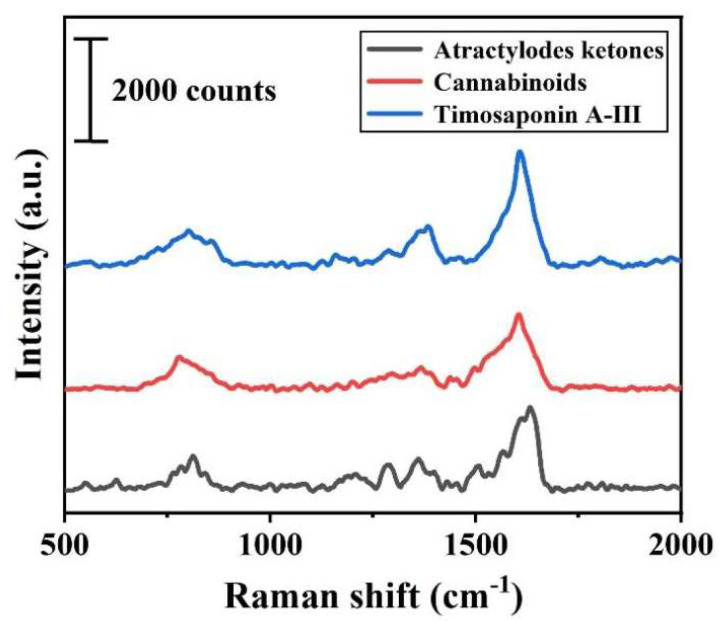
Spectroscopic analysis of three pharmacodynamic substances immobilized on Ag_30_/MW SERS substrate (all spectra correspond to a concentration of 1 × 10^−6^ M).

Figure 6 displays the spectra of the three TCM pharmacodynamic substance solutions obtained on the Ag_30_/MW SERS substrate. The Raman spectra of the three pharmacodynamic substances show distinct response signals at multiple characteristic peaks, including, but not limited to, the peak around 1648 cm^−1^. Notably, the characteristic peaks of atractylon and timosaponin A-III significantly overlap, which complicates their identification in mixtures. Therefore, we employed deep learning to address this challenge. As an advanced data analysis technique, deep learning plays a crucial role in developing a novel, highly sensitive, efficient, and accurate detection method for TCM pharmacodynamic substances by leveraging SERS technology [27]. Based on the spectral data acquired from the high-performance Ag_30_/MW SERS substrate screened in earlier stages, this study introduces a deep learning model as the core component to achieve intelligent identification. This work provides foundational validation for the application of deep learning in the field of TCM detection and establishes an intelligent identification framework suitable for the three pharmacodynamic substances.

**Figure 7:**
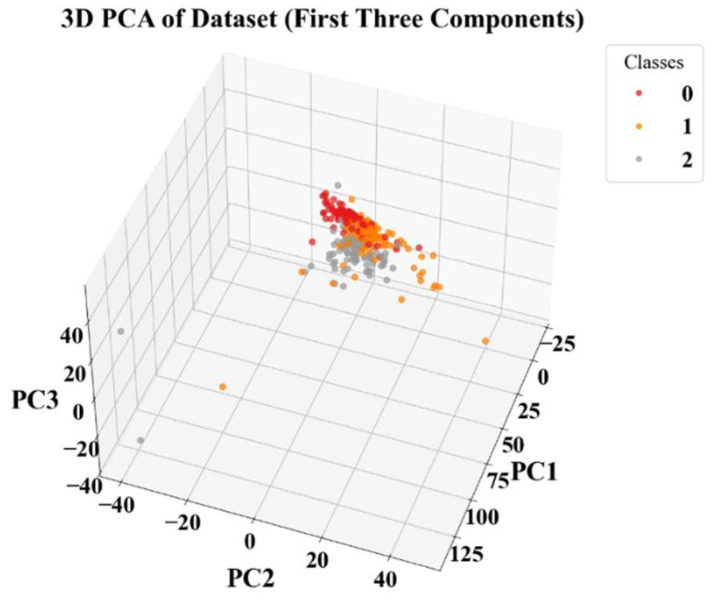
3D PCA of Dataset (First Three Components).

Given that Raman spectra contain substantial redundant information across the full wavenumber range (with many wavenumbers lacking characteristic signals), directly inputting them into a model would lead to the curse of dimensionality. Therefore, PCA was first applied for data dimensionality reduction. The first two principal components (PC1 and PC2) were extracted as the core features. These two components captured over 90% of the effective information from the original spectra, thereby reducing the model’s computational load while preserving key spectral differences among the substances. From the two-dimensional distribution plot after PCA reduction shown in Figure 7, it is evident that the sample points of the three pharmacodynamic substances exhibit partial overlap (particularly pronounced between atractylon and timosaponin A-III). This indicates that while PCA effectively compresses the data, the resulting feature space still contains complex, non-linear relationships that may challenge classical linear classifiers, especially when discriminating overlapping spectral signatures from structurally similar compounds. Even with reduced feature sets, deep learning models can learn such complex decision boundaries. This approach is supported by prior research in spectral analysis [28,29].

The processed SERS data were subsequently classified using four deep learning models: MLP, Transformer, ResNet, and DNN. By comparing the loss and accuracy curves, we evaluated the strengths and limitations of each model for classifying SERS data. This analysis ensures that the models can be effectively applied not only to the detection of low-concentration TCM pharmacodynamic substances but also to other complex data classification tasks.

**Figure 8:**
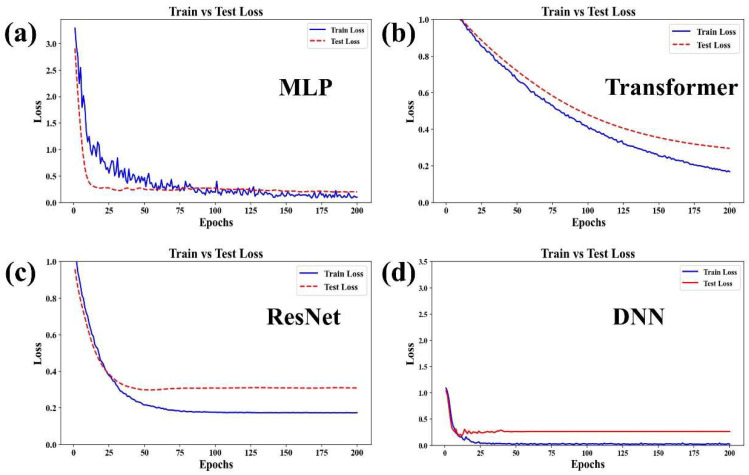
Loss curves of four deep learning models: (**a**) MLP. (**b**) Transformer. (**c**) ResNet. (**d**) DNN on the training set and test set.

As shown in Figure 8, the four panels illustrate the trends of loss values on the training set (Train Loss) and the test set (Test Loss) across training epochs for the four deep learning models (MLP, Transformer, ResNet, and DNN). Their key characteristics are summarized as follows: In MLP model, the training loss (blue line) decreased rapidly but continued to fluctuate without fully stabilizing. The test loss (red line) declined and then plateaued. Although the gap between the training and test losses gradually widened, no clear signs of overfitting were observed, indicating that the model achieved an optimal balance between fitting performance and generalization ability [30]. In the Transformer model, both the training and test losses decreased synchronously throughout the training process. In the later stages, the gap between them remained small and both tended to stabilize. The model demonstrated good convergence and a low degree of overfitting [31]. In the ResNet model, the training loss dropped quickly to near zero and remained stable. The test loss decreased but showed slight fluctuations afterward, maintaining a relatively small gap from the training loss. The model exhibited a moderate level of overfitting [32]. In the DNN model, both the training and test losses decreased rapidly. In the later epochs, they almost overlapped and stabilized at a low level. The significant gap between the training and test losses indicates clear signs of overfitting [33].

**Figure 9:**
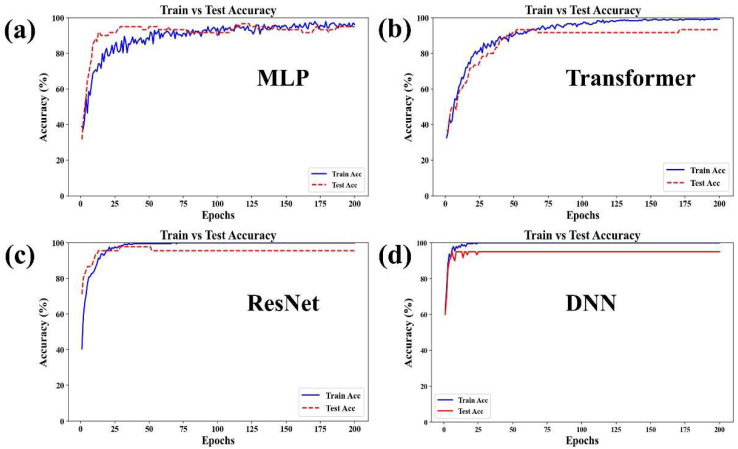
Accuracy curves of four deep learning models: (**a**) MLP. (**b**) Transformer. (**c**) Resnet. (**d**) DNN on the training set and test set.

Figure 9 and Table 2 illustrate the trends of accuracy on the training set (Train Acc) and the test set (Test Acc) across training epochs for the four deep learning models (MLP, Transformer, ResNet, and DNN). Their key characteristics are summarized below: In the MLP model, the training accuracy (blue line) increased rapidly and then stabilized at a high level with minor fluctuations. The test accuracy (red line) also rose and remained close to the training accuracy, showing only a small gap between them. This indicates mild overfitting, and the model demonstrated the best overall fitting performance. In the Transformer model, both the training and test accuracies increased synchronously. In the later stages, they stabilized with a relatively small gap between them, although the final test accuracy remained lower than the training accuracy. The model exhibited relatively good generalization ability but still showed a noticeable degree of overfitting. In the ResNet model, the training accuracy rose quickly and stabilized near 100%. The test accuracy increased but displayed slight fluctuations afterward. The gap between the training and test accuracy was smaller than that of the MLP model, indicating a moderate level of overfitting. In the DNN model, the training accuracy rapidly reached 100% and remained stable. The test accuracy also increased quickly to a level close to 100%, nearly overlapping with the training accuracy curve. However, compared to the MLP model, it demonstrated more pronounced overfitting.

**Figure 10:**
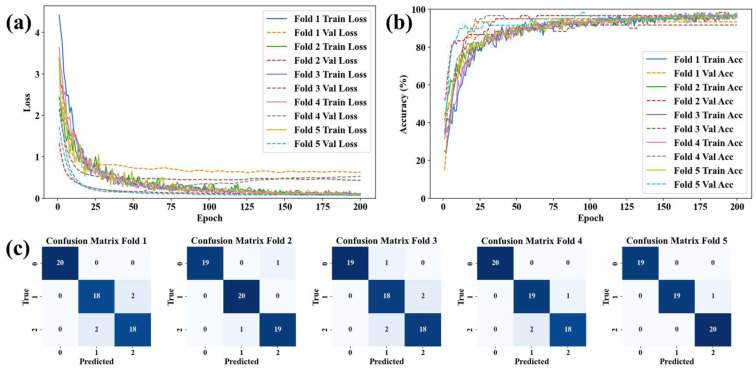
Five-fold cross-validation curves of the MLP model in the SERS medicine classification task. Each fold shows the loss and accuracy trends of the training set and validation set. (**a**) Loss curves of the training set and test set for each five-fold cross-validation; (**b**) accuracy curves of the training set and test set for each five-fold cross-validation; (**c**) confusion matrix diagram of five-fold cross-validation.

Figure 10 presents the five-fold cross-validation results of the MLP model on the SERS-based drug classification task, which can be interpreted in three parts: Firstly, in Figure 10a, the training and validation losses of all folds decrease rapidly with increasing epochs and eventually stabilize at a low level (close to 0). The nearly overlapping training and validation loss curves indicate no overfitting and good generalization ability. Secondly, in Figure 10b, the training and validation accuracies of all folds increase quickly with epochs and finally stabilize above 95% (close to 100%). The consistent alignment between training and validation accuracies further confirms the model’s stability and generalization performance. Thirdly, in Figure 10c, the confusion matrix for each fold shows: Minimal classification errors across all categories (most cells show 0 or 1 error). For instance, only 3 samples were misclassified in Fold 1. The overall classification accuracy is extremely high, confirming excellent performance in practical classification tasks.

Therefore, the deep-learning-based MLP classification framework exhibits outstanding accuracy, generalization capability, and robustness in the identification and classification of TCM pharmacodynamic substances [30]. The average validation accuracy = 0.9466 ± 0.0220 and the average validation AUC = 0.9816 ± 0.0143. It effectively extracts subtle biological information from high-dimensional spectral data, automatically performs feature extraction and classification, and consistently maintains high performance under five-fold cross-validation, confirming the stability of its architecture and the reproducibility of results. These findings suggest that the proposed MLP-driven analytical approach holds great potential for the precise analysis of complex biological samples on SERS platforms, opening a new intelligent pathway for the identification of TCM substances.

The establishment of this intelligent recognition system not only addresses the key bottlenecks in traditional detection—such as the difficulty in spectral interpretation and low identification efficiency—but also provides a practical pathway for applying deep learning in the field of TCM analysis. On the one hand, the highly sensitive spectral data acquired from the Ag_30_/MW SERS substrate supplies high-quality input features for the model, thereby minimizing identification deviations caused by signal noise. On the other hand, the “PCA dimensionality reduction and deep-learning classification” framework is directly transferable to other scenarios involving TCM pharmacodynamic substances. Only the corresponding spectral dataset needs to be replaced for rapid adaptation.

While this study focuses on demonstrating the feasibility of the proposed SERS-deep learning framework, we acknowledge that certain aspects warrant further investigation. Specifically, a direct comparison with classical machine learning classifiers and a quantitative benchmark against literature-reported detection limits would further strengthen the validation. Such comparisons are essential to contextualize the performance of the MLP model and to establish a more comprehensive evaluation framework for TCM-related SERS applications. Future work will therefore include systematic comparisons with classical algorithms and efforts to determine the limit of detection for each pharmacodynamic substance in more complex matrices. In parallel, more advanced deep learning models such as convolutional neural networks (CNNs) could be introduced to automatically extract deeper spectral features—including peak-shape variations and baseline-drift corrections—thereby offering stronger technical support for automated and intelligent TCM quality control. Furthermore, additional physicochemical characterization—such as Fourier-transform infrared spectroscopy (FT-IR) or X-ray diffraction (XRD)—could provide complementary structural validation of both the SERS substrate and the pharmacodynamic substances. Incorporating these techniques in future studies involving real TCM extracts would help confirm compound identities and substrate composition, thereby further strengthening the analytical framework. Together, these future directions aim to advance the practical utility of this approach and facilitate its translation into real-world analytical scenarios.

## 4. Conclusions

This study tackles a key technical bottleneck in the trace detection of TCM pharmacodynamic substances. We developed a high-performance SERS substrate based on a grating-like micro-nanostructure template derived from MW surfaces. By integrating deep learning technology, we achieved rapid and sensitive detection and identification of three pure pharmacodynamic substances—atractylon, cimifugin, and timosaponin A-III—which serve as models for compounds found in authentic medicinal materials. The substrate with the highest enhancement factor and optimal SERS performance, designated Ag_30_/MW, was selected as the optimal platform. It exhibits an enhancement factor of 6.47 × 10^6^ and a reproducibility with a relative standard deviation (RSD) of 7.03%, demonstrating considerable enhancement capability. Furthermore, by combining principal component analysis (PCA) with deep learning algorithms, we successfully performed dimensionality reduction and classification of complex spectral data, achieving a model accuracy of 95%. This approach overcomes the drawbacks of conventional detection methods, which are often tedious, time-consuming, and lacking in sufficient sensitivity. The completion of this research demonstrates a methodological framework with potential value for future identification and detection of pharmacodynamic substances in authentic medicinal materials, pending validation in real TCM extracts and multi-component systems.

## Figures and Tables

**Table 1:** Assignment of selected Raman peaks of R6G.

Neat R6G Experimental [22]	Experimental SERS Characteristic Peak	Band Assignment
613	611	In plane C–C–C bending
775	774	Out of plane C–H bending
1184	1187	In plane xanthenes ring deformation, C–H bending, N–H bending
1364	1361	Xanthenes ring stretching, in plane C–H bending
1512	1510	Xanthenes ring stretching, C–N stretching, C–H bending, N–H bending
1577	1572	Xanthenes ring stretching, in plane N–H bending
1651	1648	Xanthenes ring stretching, in plane C–H bending

**Table 2:** Performance Evaluation Table of Deep Learning Models.

Deep Learning Model	Loss	Accuracy	AUC
DNN	Train Loss: 0.0207	Train Accuracy: 100%	0.9829
	Test Loss: 0.2639	Test Accuracy: 95.00%	
MLP	Train Loss: 0.0980	Train Accuracy: 96.23%	0.9871
	Test Loss: 0.1988	Test Accuracy: 95.00%	
ResNet	Train Loss: 0.1740	Train Accuracy: 100%	0.9784
	Test Loss: 0.3091	Test Accuracy: 95.56%	
Transformer	Train Loss: 0.1503	Train Accuracy: 99.16%	0.9767
	Test Loss: 0.2957	Test Accuracy: 93.33%	

## Data Availability

Data underlying the results presented in this paper are not publicly available at this time but may be obtained from the authors upon reasonable request.
